# Hydrostatic reduction of intussusception in children: a single centre experience

**DOI:** 10.11604/pamj.2020.36.263.21380

**Published:** 2020-08-11

**Authors:** Kevin Emeka Chukwubuike, Obinna Chukwuebuka Nduagubam

**Affiliations:** 1 Department of Surgery, Enugu State University Teaching Hospital, Enugu, Nigeria,; 2 Department of Paediatrics, Enugu State University Teaching Hospital, Enugu, Nigeria

**Keywords:** Intussusception, hydrostatic, experience, single centre

## Abstract

**Introduction:**

intussusception is a common surgical emergency in children especially in infants. Treatment of intussusception could be non-operative or operative. Non-operative treatment (hydrostatic reduction) of intussusception is increasingly being practiced in developing countries.

**Methods:**

this was a review of our experience in the hydrostatic reduction of intussusception in children at a teaching hospital in Enugu, Nigeria. This study covered an 18-months period, October 2017 to March 2019. Patients on presentation were resuscitated, appropriate investigations done and prepared for surgery before the hydrostatic reduction (using normal saline) was carried out. Patients with features of peritonitis and marked abdominal distension were excluded from hydrostatic reduction.

**Results:**

twenty patients who had 21 episodes of intussusception were analyzed. One patient had a recurrence that necessitated repeat hydrostatic reduction. Eighty percent of the patients were male. The mean and peak age of the patients was 8 months and 6 months respectively. Significant number of the patients presented after 48 hours of onset of their symptoms. Abdominal pain was the predominant presenting symptom. Twenty percent and fifteen percent of the patients had a history of preceding gastrointestinal and respiratory infections preceding the intussusception respectively. Ileocolic intussusception was the most common type and the most distal end of the intussusception was at the transverse colon. Hydrostatic reduction was successful in 13 patients (65%).

**Conclusion:**

hydrostatic reduction is a simple and effective method of treatment of intussusception. However, early presentation and proper patient selection is necessary for optimal outcome.

## Introduction

Intussusception is the telescoping or invagination of a portion of the intestine into an adjacent segment. The portion that invaginates is called intussusceptum while the portion that receives the intussusceptum is the intussuscipiens. It is one of the most common causes of intestinal obstruction especially in infants and a common cause of pediatric abdominal surgical emergency [1-3]. One to four per 2000 children is the reported incidence of intussusception [4]. In most cases, intussusception in children is known to be idiopathic, without any underlying disease. However, idiopathic intussusception can also occur after respiratory or gastrointestinal viral infections [5]. Several types of intussusception have been described; the ileocolic type being the most common [6]. The classic symptoms of intussusception include abdominal pain, passage of red currant stool and abdominal mass. This triad is present in less than a quarter of children [7]. Ultrasound is the investigation of choice for intussusception because ultrasound has high specificity and sensitivity of nearly 100% for intussusception [8]. The role of abdominal X-ray lies in the detection of free air in the peritoneal cavity which indicates bowel perforation [1]. Computed tomography (CT) scan can also make a diagnosis of intussusception but the high risk of irradiation exposure makes CT scan unsuitable in children. Management of intussusception should be multidisciplinary involving the pediatric surgeon, pediatrician, radiologist, anesthetist and pediatric nurses. Treatment of intussusception could be non-operative or operative. Non-operative treatment is achieved by the use of air, oxygen (pneumatic) or barium, water, normal saline, Ringer’s lactate, Hartmann’s solution (hydrostatic), as enema, for the reduction of intussusception. This study was conducted to evaluate our experience, in terms of patients’ demography, clinical presentation, complications and outcome, in the hydrostatic reduction of intussusception in children using normal saline.

## Methods

This was a clinical study of children who presented with intussusception at the pediatric surgery unit of a teaching hospital in Enugu south-eastern Nigeria. This study covered an 18-months period, October 2017 to March 2019. The exclusion criteria were the presence of signs of peritonitis, marked abdominal distension and radiological evidence of free peritoneal air. Patients older than 15 years were also excluded from the study. Commercially available, real time ultrasound scanner with 5-MHz linear transducer (TITAN; Sonosite Inc, Bothell, WA, USA) was used for diagnosing intussusception and monitoring reduction of the intussusception. Both transverse and longitudinal views were used for confirming target sign and pseudokidney sign respectively. The procedure, its advantages and probable outcome were explained to the parents or caregiver and a written informed consent was obtained from them. Vascular access was established and intravenous fluid commenced to correct fluid and electrolyte deficits. Nasogastric tube, urethral catheter were also passed and broad spectrum antibiotics given. Adequate hydration was indicated by an hourly urine output of 0.5 to 1 milliliter (ml) per kilogram. Investigations such as full blood count, serum electrolyte, urea and creatinine, blood grouping and cross-matching were done. All the preparations necessary for surgery were made as a safeguard in case surgery became necessary in the event of failed reduction or a complication arising during the reduction. The child was put on left lateral position and a size 20F Foley’s catheter inserted into the rectum after lubrication with KY jelly.

The balloon of the catheter was inflated by injecting 30 mls of water. One liter of normal saline (pre-warmed to normal body temperature) was suspended 100 centimeter above the bed level and connected to the catheter via a fluid line. The normal saline was allowed to run freely into the rectum under the effect of gravity. Gradual distension of the colon and retrograde movement of the intussusception towards the caecum were monitored by real time ultrasound. Criteria for successful reduction include disappearance of the intussusception and passage of fluid and air bubbles from the caecum into the terminal ileum. A maximum of 3 attempts at hydrostatic reductions were made with each episode lasting for 3 minutes. Sudden increase in fluid in the peritoneal cavity and simultaneous loss of fluid from the bowel indicates bowel perforation. Bowel perforation is an absolute indication for surgery. After the hydrostatic reduction, the catheter was removed and the saline drained out through the anus. Clinical condition of the patient was monitored throughout the procedure. Data collected included patients’ age, gender, presenting symptoms, duration of symptoms before presentation, associated respiratory/gastrointestinal infection (if any), outcome of treatment and complications of hydrostatic reduction. Ethical approval was obtained from the Ethics and Research Committee of the Teaching Hospital. Statistical Package for Social Science (SPSS) version 21 was used for data entry and analysis. Data were expressed as percentages, mean and range.

## Results

**Patients’ demography:** during the study period, 56 children with 57 episodes of intussusception were managed. Thirty five patients presented with features of peritonitis or marked abdominal distension and were taken to theatre for laparotomy. Only 20 patients, who had 21 episodes of intussusception that had hydrostatic reduction, were analyzed. One patient had recurrent intussusception 24 hours after the initial reduction. There were 16 males (80%) and 4 females (20%), with a male to female ratio of 4:1. The age range of the patients was 5 months to 11 months with a mean age of 8 months. The peak age of occurrence of intussusception in our patients was 6 months. None of our patients was up to the age of 12 months. Nine patients (45%) presented after 48 hours of onset of their symptoms, eight patients (40%) between 24 to 48 hours while three patients (15%) presented within 24 hours of onset of symptoms (Table 1).

**Table 1:** demography of the patients

**Gender**	
Male	16(80%)
Female	4(20%)
Age range	5-11 months
Mean age	8 months
Peak age	6 months
**Duration of symptoms before presentation**	
More than 48 hours	9 patients (45%)
24 to 48 hours	8 patients (40%)
Within 24 hours	3 patients (15%)

**Presenting symptoms:** the patients presented with abdominal pain, bilious vomiting, passage of red currant jelly stool and abdominal mass, in various combinations. Abdominal pain was the predominant in 11 patients (55%). Others are shown in Table 2.

**Table 2:** presenting symptoms

Symptom	Number	Percentage
Abdominal pain	11	55
Passage of red currant jelly stool	4	20
Bilious vomiting	3	15
Abdominal mass	2	10

**Preceding respiratory or gastrointestinal infection:** twelve patients (60%) had no history of preceding respiratory or gastrointestinal infections. Four patients (20%) had gastrointestinal infection evidenced by diarrhea and non-bilious vomiting preceding the onset of intussusception. Three patients (15%) had respiratory infection evidenced by cough and nasal discharge prior to the commencement of the abdominal symptoms that signifies intussusception. One patient (5%) had symptoms of both respiratory and enteral infections.

**Type of intussusception:** ultrasound evaluation of the patients before the hydrostatic reduction showed ileocolic intussusception in 16 patients (80%) and ileo-ileal intussusception in 4 patients (20%). There was no case of colo-colic intussusception (Table 3).

**Table 3:** types of intussusception

Type of intussusception	Number	Percentage
Ileocolic	16	80
Ileo-ileal	4	20
Colo-colic	None	0

**Location of the distal end of the intussusceptum:** in eleven patients (55%), the distal end of the intussusceptum was located at the transverse colon, four patients (20%) at the ascending colon, three patients (15%) at the descending colon and two patients (10%) at the sigmoid colon.

**Outcome of treatment:** hydrostatic reduction was successful in 13 patients (65%) while in 7 patients (35%) it was unsuccessful. One patient who had a successful reduction had a recurrence that was treated with a repeat hydrostatic reduction. Of the seven patients that had failed reduction, there was one case of bowel perforation. This necessitated an emergency laparotomy and a right hemicolectomy was done.

## Discussion

Intussusception was first described by Paul Barbette of Amsterdam in 1692. In 1876, Harald Hirschsprung made the first attempt at hydrostatic reduction of intussusception using water. The sonographic features of intussusception were described in 1977 [9,10]. Kim and his colleagues described the first successful sonographic guided hydrostatic reduction in 1982 [11]. While hydrostatic reduction of intussusception is well established in developed countries, its practice in developing countries is shaky due to late presentation, lack of facilities and appropriate expertise [12,13]. The male dominance recorded in the current study is consistently observed in many other reports too [8,14,15]. The mean age of our patients is similar to that Khorana *et al*. but varies with that of other studies of its nature [4,8,10,14]. The reason for these differences is not clear but may be explained by geographical location, dietary patterns and age of weaning of the infants. Most of our patients presented after 48 hours of onset of their symptoms. This is in agreement with the findings of other researchers [16,17]. It is noteworthy that this late presentation is mostly seen in developing countries where there is lots of poverty and ignorance. The finding of abdominal pain as the most common presenting symptom of intussusception is in line with the reports of other studies [14,18]. However, other studies reported vomiting and abdominal mass as the most common symptoms [4,15,19].

The difference in presenting symptoms may be due to stage of the disease or time of presentation to the hospital. Abdominal pain in intussusception presents as a sudden onset colicky pain and this makes the child to draw the knees towards the chest. The rate of virus detection is high in the stool of patients that have intussusception [20]. There are suggestions that occurrence of intussusception has to do with viral infections of the respiratory tract or gastrointestinal tract which eventually causes hyperplasia of the lymphoid tissues such as the peyer’s patches. The enlarged peyer’s patches may subsequently be a lead point for intussusception [20]. Okimoto *et al*. and Mansour *et al*. in their separate studies reported a positive association of antecedent diarrheal or respiratory illness with increased risk of intussusception [21,22]. In the present study, ileocolic (idiopathic) type of intussusception was the most common type. This is consistent with the observation of most series [7,14,23]. Other types of intussusception such as ileoileal, colocolic, jejunoileal, jejunojejunal can also occur in children but they are far less common than ileocolic type. When a pathologic lead point is involved, any part of the bowel may be involved in intussusception. In about 5-6% of intussusceptions in children, there are pathologic lead points which are due to focal masses or diffuse bowel wall abnormality [24].

In decreasing order of frequency, the most common pathologic lead points are Meckel’s diverticulum, duplication cyst, polyp and lymphoma [25]. Mensah and his colleagues reported transverse colon as the most common location of intussusception in their patients [8]. This is also what we found in the current study. However, the location of intussusception depends on length of the mesentery and time of presentation to the hospital. Late presenting cases may present as prolapsing intussusception through the anus [26]. Our success rate of 65% is comparable to other published reports [4,8,10,15]. However, other workers reported higher success rates [1,14]. Chalya and his colleagues treated all their patients surgically. The differences in success rate may be attributable to patient selection, age of the patients, material used for the hydrostatic reduction or expertise of the surgeon/sonologist. This study is not without limitations. This study has a small sample size. A larger number of cases would have availed better analysis and basis for critical comparison with other published larger series. Secondly, single centre series cannot be generalized as the outcome may be affected by the skill of the practitioner.

## Conclusion

Hydrostatic reduction of intussusception is a simple and effective method for the treatment of intussusception. Success rate of 65% is good considering the morbidity that is associated with operative treatment. However, early presentation and proper patient selection is necessary for optimal outcome. Where the expertise is available, we recommend hydrostatic reduction as the first modality of treatment for intussusception.

### What is known about this topic

- Treatment of intussusception is mostly surgical;
- Hydrostatic reduction is not commonly practiced in developing countries.

### What this study adds

- This study creates the awareness about the effectiveness of hydrostatic reduction of intussusception in a developing country;
- Selected groups of patients can benefit from this modality of treatment.
